# High Salivary Malondialdehyde Levels Are Associated with Periodontitis Independently of Other Risk Factors

**DOI:** 10.3390/jcm14092993

**Published:** 2025-04-26

**Authors:** Leonardo Lorente, Esther Hernández Marrero, Pedro Abreu González, Angel Daniel Lorente Martín, Agustín F. González-Rivero, María José Marrero González, Carmen Hernández Marrero, Olga Hernández Marrero, Alejandro Jiménez, Cándido Manuel Hernández Padilla

**Affiliations:** 1 Intensive Care Unit, Hospital Universitario de Canarias, Ofra s/n. La Laguna, 38320 Santa Cruz de Tenerife, Spain; 2 Clínica Dental Cándido, Plaza San Cristóbal 35, La Laguna, 38320 Santa Cruz de Tenerife, Spain; esther@clinicadentalcandido.com (E.H.M.); mariajose@clinicadentalcandido.com (M.J.M.G.); carmen@clinicadentalcandido.com (C.H.M.); olga@clinicadentalcandido.com (O.H.M.); candido@clinicadentalcandido.com (C.M.H.P.); 3 Unit of Physiology, Department of Basic Medical Sciences, Faculty of Medicine, University of La Laguna, Ofra s/n. La Laguna, 38320 Santa Cruz de Tenerife, Spain; pabreu@ull.edu.es; 4 Department of Odontology, Faculty of Medicine, CEU San Pablo University, Avenida Montepríncipe s/n. Boadilla del Monte, 28660 Madrid, Spain; daniellorentemartin@gmail.com; 5 Laboratory Department, Hospital Universitario de Canarias, Ofra s/n. La Laguna, 38320 Santa Cruz de Tenerife, Spain; afgonriv@gmail.com; 6 Research Unit, Hospital Universitario de Canarias, Ofra s/n. La Laguna, 38320 Santa Cruz de Tenerife, Spain; ajimenezsosa@gmail.com

**Keywords:** oxidation, salivary, malondialdehyde, periodontitis, gingivitis

## Abstract

**Background/Objectives:** Patients with periodontitis have been found to have higher salivary concentrations of malondialdehyde, a biomarker of lipid hyperoxidation, compared to healthy subjects. However, the association between salivary malondialdehyde levels and periodontitis, independently of other risk factors, has not been analyzed. Therefore, the novel objective of our study was to explore this possible association by performing a logistic regression analysis. **Material and Methods:** This observational, prospective study was carried out in a private dental practice. Salivary malondialdehyde levels were measured in subjects with periodontitis (characterized by periodontal tissue loss) and in subjects without periodontitis (either periodontally healthy or with localized gingivitis in <30% of sites). A multivariate regression analysis was carried out to determine the factors associated with periodontitis. Variables with a *p*-value ≤ 0.05 in the comparison between subjects with and without periodontitis were included in the regression analysis. **Results:** A total of 119 subjects were included, 63 with periodontitis and 56 without (35 periodontally healthy subjects and 21 patients with localized gingivitis). In the multiple binomial regression analysis, salivary malondialdehyde levels > 0.77 nmol/mL were identified as a predictor of periodontitis (*p* = 0.03), after controlling for age (*p* < 0.001), diabetes mellitus (*p* = 0.81), arterial hypertension (*p* = 0.43), smoking status (never smoked *p* = 0.08), and cardiovascular disease (*p* = 0.88). **Conclusions:** To our knowledge, this is the first study to report that salivary malondialdehyde levels are associated with periodontitis, independently of other known risk factors.

## 1. Introduction

Periodontitis, a chronic inflammatory disease of the periodontium, represents a significant public health problem due to its widespread global prevalence and economic impact [1,2,3,4]. In periodontitis, different pathophysiological pathways are activated, such as inflammation [2,3,4], oxidation [5,6,7], and programmed cell death [8].

The clinical diagnosis of periodontitis can be interfered with by certain factors such as the pressure applied with the periodontal probe in the crevice, limited mouth openings, and discomfort during the examination [2].

Thus, some salivary biomarkers have been suggested that could help in the diagnosis of periodontitis and the classification of periodontitis severity [2,3,4], although they are not being used in clinical practice. In this respect, different biomarkers in saliva have been found in higher concentrations in patients with periodontitis than in healthy subjects as interleukin (IL)-1β, S100A8, S100A9, S100A12, matrix metalloproteinase (MMP)-8, and hepatocyte growth factor (HGF), colony-stimulating factor-1 (CSF-1) [2], interleukin (IL)-6, tumor necrosis factor-alpha (TNF-alpha), osteoprotegerin [3], and prostaglandin E2 (PGE2) [4], malondialdehyde (MDA), nitric oxide (NO), total oxidant status (TOS), and 8-hydroxy-deoxyguanosine [5].

The increased production of reactive oxygen species (ROS) can induce multiple adverse effects, such as lipid, protein, and nucleic acid oxidation [5,6,7] as well as the activation of programmed cell death [5,6,7]. Periodontal inflammation is associated with high levels of malondialdehyde (a biomarker of lipid hyperoxidation) in saliva.

Malondialdehyde is an end-product that appears during lipid peroxidation, specifically due to phospholipid degradation of the cellular membrane. Due to the action of phospholipase-A_2_, arachidonic acid is produced. Afterwards, arachidonic acid is attacked by mitochondrial ROS (mainly by hydroxyl radical), forming lipid endoperoxide. Subsequently, due to the spontaneous rupture of lipid endoperoxide, malondialdehyde in the intracellular space is formed. Finally, malondialdehyde is released into the extracellular space and will appear in blood and saliva [6].

A meta-analysis published by Chen et al. in 2019, which included 14 articles and 931 subjects (474 with and 457 without periodontitis), reported higher salivary concentrations of malondialdehyde in subjects with periodontitis than in periodontally healthy subjects in the bivariate analysis [5]. In the meta-analysis published by Mohideen et al. in 2023, which included 10 articles and 763 subjects (393 with and 370 without periodontitis), higher salivary concentrations of malondialdehyde in subjects with periodontitis were reported than in healthy subjects in the bivariate analysis [6]. Those 10 articles and 763 subjects were also included in the meta-analysis published by Chen et al. In 2019 [5]. In addition, no new studies were included in the meta-analysis by Mohideen et al. with respect to the meta-analysis by Chen et al. [5]. In addition, some studies have found an association between malondialdehyde salivary levels and periodontitis severity [9,10,11,12].

Different risk factors have been associated with the development of periodontitis, such as age [13], sex [14], obesity [15], diabetes mellitus [16], systemic lupus erythematosus and rheumatoid arthritis [17], arterial hypertension [18], dental hygiene, consumption of tobacco and alcohol, immunosuppression and oral cancer [19].

However, the potential association of salivary malondialdehyde levels and periodontitis, independent of other risk factors, has not been analyzed. We hypothesize that there is an association between salivary malondialdehyde levels and periodontitis regardless of other risk factors. Therefore, the main and novel objective of our study was to explore this possible association by performing a logistic regression analysis.

As the clinical diagnosis of periodontitis can be interfered with by some factors [2], the use of salivary biomarkers could help in the diagnosis of periodontitis [2,3,4]. Therefore, one secondary objective of this study was to explore the potential role of salivary malondialdehyde levels in the diagnosis of periodontitis, performing a receiver operating characteristic (ROC) analysis.

## 2. Methods

### 2.1. Design and Subjects

The Clinical Research Ethics Committee of the Hospital Universitario de Canarias approved the study protocol (CHUC_2023_138). Informed consent was obtained from each subject prior to inclusion in this study.

This prospective and observational study was developed at Clínica Dental Cándido in La Laguna, Tenerife, Spain. This study included subjects with periodontitis (loss of periodontal tissue) and subjects without periodontitis (periodontal health or localized gingivitis in <30% of sites). We used current internationally accepted criteria for the definitions of periodontal health, gingivitis, and periodontitis, and for the classification of periodontitis severity [20]. Subjects younger than 18 years and lactating females were excluded.

### 2.2. Definitions

Clinical periodontal health was defined as the absence of bleeding on probing or its presence in less than 10% of sites, along with the absence of clinical interproximal attachment loss and bone loss due to periodontitis.

Localized gingivitis was defined as the presence of bleeding in 10–30% of sites, with the absence of clinical interproximal attachment loss and bone loss due to periodontitis.

Periodontitis was defined as the bone loss or clinical interproximal attachment loss. In some cases, tooth loss may have occurred due to periodontitis. The severity of periodontitis was determined based on the following criteria: (1) Clinical interproximal attachment loss: Stage I <3 mm, Stage II 3–4 mm, Stage III or IV ≥5 mm. (2) Radiographic bone loss: Stage I when was coronal third (<15%), Stage II when affecting 15–33%), Stage III or IV when there was extension to the middle or apical third of the root. (3) Tooth loss: Stage I or II when none was, Stage III when ≤4 teeth were lost, Stage IV when ≥5 teeth were lost.

We used periodontal probes PCP 12 of Hu Friedy (Chicago, IL, USA). All the teeth of each subject were probed in 6 surfaces: mesial, central, and distal, both on the vestibular (external) and lingual (internal) surfaces. We used orthopantomography to make a radiological diagnosis.

### 2.3. Variables Recorded

The following variables were recorded: age, sex, diabetes mellitus, arterial hypertension, cardiovascular disease, hypercholesterolemia, oral cancer, consumption of tobacco, coffee, tea, alcohol and drugs, dental hygiene, body mass index (BMI) (kg/m^2^), obesity (BMI ≥ 30 kg/m^2^), immunosuppressive therapy, radiotherapy, chemotherapy, methotrexate, loss of clinical interproximal attachment, radiographic bone loss and tooth loss due to periodontitis.

Samples of unstimulated whole saliva were collected using the technique described by Navazesh [21]. Samples were taken in the morning from 8 to 10 a.m. to avoid possible influences of the circadian rhythm on the concentrations of biomarkers in saliva. Subjects were instructed to fast overnight and refrain from smoking, drinking, or brushing their teeth for 2 h before the session. They rinsed their mouths thoroughly 3 times with 10 mL of deionized water. They were then seated comfortably with their eyes open, heads tilted slightly forward, and rested for 30 min, trying to minimize orofacial movements. Saliva was allowed to accumulate on the floor of the mouth and was collected into a container over a 30 min period, without swallowing. The collected samples were then centrifuged at 3000 rpm for 10 min at 24 °C to remove debris and cells. The supernatant was then pipetted into Eppendorf tubes and stored immediately in a freezer at −80 °C until the analytical determinations were performed.

Some subjects included in this study were previously part of other publications by our research team in which salivary levels of nitrites [22] and uric acid [23] were determined. In the current study, salivary malondialdehyde levels were determined.

### 2.4. Salivary Malondialdehyde Level Analysis

Salivary malondialdehyde levels were measured using the thiobarbituric acid-reactive substance (TBARS) method described by Kikugawa et al. [24]. To minimize potential interferences, we used the methods described by Hodges et al. [25] and Valenzuela [26]. The samples were run in duplicate. The internal positive control was authentic malondialdehyde as standard (Merck-Millipore Sigma, Madrid, Spain). Each assay included 10 malondialdehyde standards. The assay had a detection limit of 0.079 nmol/mL, an intra-assay coefficient of variation of 1.82%, and an inter-assay coefficient of variation of 4.01%. Salivary malondialdehyde concentrations were expressed in nmol/mL.

### 2.5. Statistical Methods

Statistical analyses were performed using SPSS 17.0 (SPSS Inc., Chicago, IL, USA) and LogXact 4.0 (Cytel Software Corporation, Cambridge, MA, USA). Kruskal–Wallis test was used to determine whether there was a possible difference in salivary malondialdehyde concentrations between subject groups.

The comparison between subject groups in categorical variables was carried out using the Chi-square test, while continuous variables were analyzed using the Mann–Whitney U-test. The potential association between salivary malondialdehyde levels and periodontitis severity was evaluated using Spearman’s rho correlation coefficient.

Receiver operating characteristic (ROC) analyses were carried out using the diagnosis of periodontitis and salivary malondialdehyde levels. Sensitivity, specificity, positive and negative likelihood ratios, positive and negative predictive values, and their 95% confidence interval (CI) were reported for the salivary malondialdehyde threshold > 0.77 nmol/mL, selected based on the Youden J index [27].

Multiple binomial logistic regression was used to estimate the association between salivary malondialdehyde levels > 0.77 nmol/mL and periodontitis, adjusting for age, arterial hypertension, and smoking status (never smoked). Variables with a *p*-value ≤ 0.05 in the comparison between subjects with and without periodontitis were included in the regression analysis.

## 3. Results

A total of 119 subjects were included, 63 with periodontitis and 56 without (35 periodontally healthy subjects and 21 patients with local gingivitis). Table 1 shows the description of periodontal status and salivary malondialdehyde levels for each periodontal state group.

Patients with periodontitis compared to subjects without periodontitis had a higher prevalence of arterial hypertension (*p* = 0.001), a higher proportion of never having smoked (*p* < 0.001), higher malondialdehyde salivary levels (*p* < 0.001), and were older (*p* < 0.001). In our cohort, there were no subjects with oral cancer, chemotherapy, drug consumption, or lack of dental hygiene (toothbrushing). There were no significant differences between subjects with and without periodontitis in sex, personal history of diabetes mellitus, cardiovascular disease, hypercholesterolemia, rheumatoid arthritis, systemic lupus erythematosus, immunosuppressive therapy, radiotherapy, methotrexate, body mass index, obesity, or consumption of coffee, tea, or alcohol (Table 2).

We have found a positive association between salivary malondialdehyde levels and age (rho = 0.27; *p* = 0.004). However, we have not found significant differences in salivary malondialdehyde levels according to sex (*p* = 0.96), diabetes mellitus (*p* = 0.21), arterial hypertension (*p* = 0.61), rheumatoid arthritis (*p* = 0.38), systemic lupus erythematosus (*p* = 0.20), cardiovascular disease (*p* = 0.95), hypercholesterolemia (*p* = 0.55), coffee (*p* = 0.57), never smoker (*p* = 0.90), tea (*p* = 0.06), alcohol (*p* = 0.31), obesity (*p* = 0.97), immunosuppressive therapy (*p* = 0.54), radiotherapy (*p* = 0.43) or methotrexate (*p* = 0.15).

A significant association was found between salivary malondialdehyde levels and the severity of periodontitis (rho = 0.40; *p* < 0.001).

**Figure 1:**
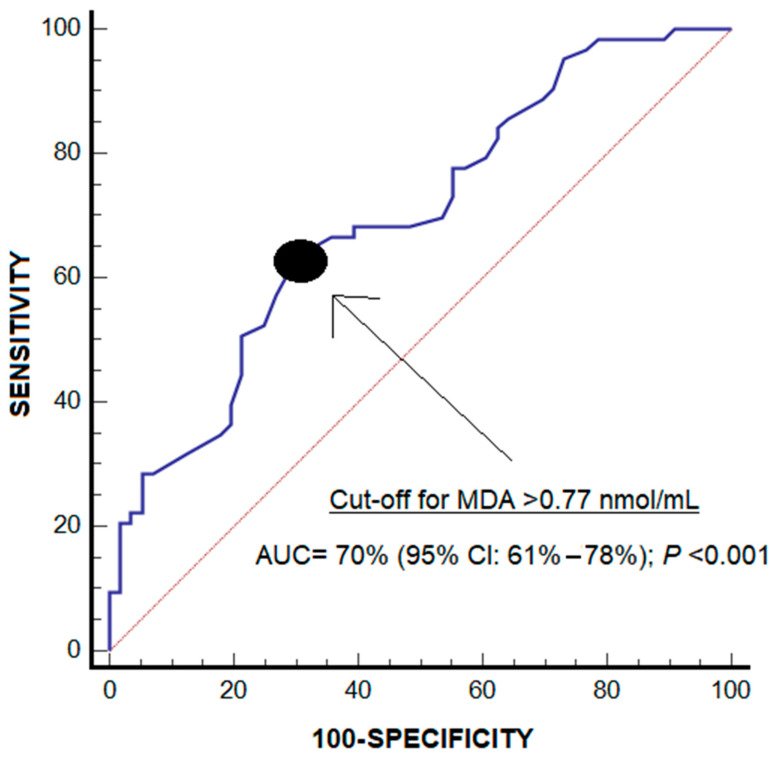
Receiver operating characteristic analysis using malondialdehyde salivary levels for diagnosis of periodontitis.

The area under the curve for the diagnosis of periodontitis by salivary malondialdehyde levels was 70% (95% CI = 61–78%; *p* < 0.001) (Figure 1). The malondialdehyde levels > 0.77 nmol/mL showed a sensitivity of 63% (50–75%), a specificity of 70% (56–81%), a positive likelihood ratio of 2.1 (1.4–3.2), a negative likelihood ratio of 0.5 (0.4–0.8), a positive predictive value of 70% (60–79%) and a negative predictive value of 63% (54–71%) for the diagnosis of periodontitis.

In the multiple logistic regression analysis, salivary malondialdehyde levels > 0.77 nmol/mL were associated with periodontitis (*p* = 0.03), controlling for age, arterial hypertension, and never smoking (Table 3).

## 4. Discussion

The findings of our study are consistent with the results of other previous studies showing higher salivary malondialdehyde levels in patients with periodontitis than in healthy oral subjects [5,6]. In addition, the novel finding of our study was that there is an association between salivary malondialdehyde levels and periodontitis independently of other known risk factors.

In the meta-analysis by Chen et al. of 2019 (including 14 articles and 931 subjects, 474 with and 457 without periodontitis), higher salivary concentrations of malondialdehyde in subjects with periodontitis than in periodontally healthy subjects were found [5]. In most of the studies included in the meta-analysis by Chen et al. were found higher salivary malondialdehyde levels were found in patients with periodontitis than in healthy controls [28,29,30,31,32,33,34,35,36,37]. However, in a few studies included in that meta-analysis, no significant differences were found in salivary malondialdehyde levels between patients with periodontitis and healthy controls [38,39,40]. In addition, in one study with 19 subjects, lower salivary malondialdehyde levels were found in patients with periodontitis compared to healthy controls [41].

In the meta-analysis by Mohideen et al. in 2023 (including 10 articles and 763 subjects, 393 with and 370 without periodontitis), higher salivary concentrations of malondialdehyde in subjects with periodontitis than in periodontally healthy subjects were found [6]. Those 10 articles were also included in the meta-analysis published by Chen et al., and no new studies were included in the meta-analysis by Chen et al. [5].

In addition, three studies that were not included in any of the meta-analyses also reported higher salivary concentrations of malondialdehyde in subjects with periodontitis than in healthy subjects [42,43,44]. In the study by Altıngöz et al., 120 subjects (60 with periodontitis and 60 healthy subjects) were included [42]. In the study by Gautam et al., 112 subjects (56 with periodontitis and 56 healthy subjects) were included [43]. In the study by Veljovic et al., 50 subjects were included (30 with periodontitis and 20 healthy subjects) [44].

Other variables that were associated with periodontitis in our study were age and never smoking, findings that have also been reported previously [13,19]. However, we found no other variables associated with periodontitis, possibly due to the relatively small sample size of our study.

We have found an association between salivary malondialdehyde levels and age; but we have not found significant differences in salivary malondialdehyde levels according to sex, diabetes mellitus, arterial hypertension, rheumatoid arthritis, systemic lupus erythematosus, cardiovascular disease, hypercholesterolemia, coffee, never smoker, tea, alcohol, obesity, immunosuppressive therapy, radiotherapy or metrotexate. It is possible that other variables that we have not registered could influence salivary malondialdehyde levels. However, the only variable associated with salivary malondialdehyde levels (age) was introduced in the regression analysis, and we found that salivary malondialdehyde and age were independently associated with periodontitis in the multiple logistic regression analysis. Thus, we think that with those findings, the bias risk could have been mitigated. This finding of our study about the positive association between salivary malondialdehyde levels and age has been previously described [45].

Another finding of our study was the positive correlation between salivary malondialdehyde levels and the severity of periodontitis, which was also found in previous studies [9,10,11,12].

All together, these results indicate that alteration of the oxidative state occurs in periodontitis. High salivary malondialdehyde levels found in patients with periodontitis could represent an increase in lipid peroxidation. Increased ROS production may contribute to high salivary malondialdehyde levels found in patients with periodontitis. Those aspects could be related to the periodontal inflammation in periodontitis.

Previous studies by our research team also found that malondialdehyde levels could be used as a biomarker of severity in patients with different diseases such as sepsis [46], traumatic brain injury [47], cerebral infarcts [48] or spontaneous intracerebral hemorrhage [49] and hepatocellular carcinoma undergoing liver transplantation [50]. We carried out this study due to the fact that we found in our previous studies that malondialdehyde levels could be used as a severity biomarker in determined patients, and we hypothesized that there could be an association between salivary malondialdehyde levels and periodontitis regardless of other risk factors.

The administration of agents that modulate the antioxidant and oxidant state could be a promising treatment for patients with severe periodontitis. In fact, treatment with melatonin has been shown to improve periodontal disease in some patients. Besides its antioxidant effects, melatonin also exerts anti-inflammatory and anti-apoptotic properties [51,52,53,54]. A systematic review by Balaji et al. analyzed the use of melatonin in the management of periodontitis, including four studies on topical (orobase cream) and eight with systemic (oral tablet) melatonin administration [53]. All four studies using topical melatonin administration reported improvements in periodontal disease. Only two of eight studies using oral melatonin administration reported periodontal parameters, and improvements in periodontal parameters were reported in those two studies using oral melatonin administration. Some studies also found a reduction in inflammatory biomarkers (C-reactive protein, tumor necrosis factor alpha, interleukin-6) in saliva and blood with the administration of topical and oral melatonin. However, only one study by Zare et al. assessed the oxidative state [54], reporting that oral melatonin reduced serum malondialdehyde levels and increased total antioxidant capacity.

The diagnosis of periodontitis is performed by clinical and radiological examination; however, the determination of salivary malondialdehyde concentrations could help in the diagnosis of periodontitis according to the results of our ROC analysis. The clinical diagnosis of periodontitis can be interfered with by certain factors such as the pressure applied with the periodontal probe in the crevice, limited mouth opening, and discomfort during the exploration [2]. Thus, the use of salivary biomarkers could help in the diagnosis of periodontitis and periodontitis severity classification [2,3,4]. It is considered that an AUC less than 0.7 is sub-optimal performance, from 0.70 to 0.80 is considered good, and a value of 0.80 or above is excellent [55,56]. Thus, the AUC of 0.7 that we found in our study is within the limit to be considered good. However, it would be interesting to conduct more studies to confirm our findings. In addition, assessing salivary malondialdehyde concentrations could help assess the saliva antioxidant status of subjects. The use of mouth creams with antioxidant agents may be suggested for individuals with high salivary malondialdehyde concentrations to help prevent or delay the development of periodontitis or to help prevent or delay its progression to more severe forms or to try to reverse its severity. As is logical, before taking this step, studies with mouth creams with antioxidant agents should first be carried out in animal models and then in humans.

We would like to acknowledge some limitations in our study. First, malondialdehyde levels were not determined in other biological samples (like blood or gingival crevicular fluid). Second, a larger sample size would have allowed us not to include patients with local gingivitis in the category of subjects without periodontitis. Third, we have not calculated the sample size; however, it was sufficient to find an association between salivary malondialdehyde concentrations and periodontitis independently of other risk factors in the regression analysis. Previously, higher salivary concentrations in subjects with periodontitis than in periodontally healthy subjects in the bivariate analysis have been reported [5,6]. However, the independent association of salivary malondialdehyde levels and periodontitis in a regression analysis has not previously been reported. Therefore, the novel objective of our study was to explore this possible association by performing a logistic regression analysis. Thus, the association between salivary malondialdehyde concentrations and periodontitis independently of other risk factors in the regression analysis that we found in our study represents the main novel finding of our study. Despite the limitations of our study, this is the first to report such findings.

## 5. Conclusions

In conclusion, to our knowledge, this is the first study reporting that salivary malondialdehyde levels are associated with periodontitis, independently of other known risk factors.

## Figures and Tables

**Table 1:** Description of periodontal state in all subjects of this study.

Variables	Total (n = 119)	Subjects Without Periodontitis (n = 56)	Subjects with Periodontitis (n = 63)	Malondialdehyde (nmol/mL)- Median (*p* 25–75)	*p*-Value
					0.001
Periodontal health—n (%)	35 (29.4)	35 (62.5)	0	0.71 (0.58–0.80)	
Localized gingivitis—n (%)	21 (17.6)	21 (37.5)	0	0.72 (0.65–0.95)	
Periodontitis stage I—n (%)	17 (14.3)	0	17 (27.0)	0.78 (0.64–1.17)	
Periodontitis stage II—n (%)	25 (21.0)	0	25 (39.7)	0.80 (0.66–0.93)	
Periodontitis stage III—n (%)	14 (11.8)	0	14 (22.2)	0.86 (0.81–1.09)	
Periodontitis stage IV—n (%)	7 (5.9)	0	7 (11.1)	0.89 (0.74–1.27)	

The comparison between subject groups in salivary malondialdehyde levels was carried out using Mann–Whitney U-test.

**Table 2:** Comparisons between subjects with and without periodontitis.

Variables	Subjects Without Periodontitis (n = 56)	Subjects with Periodontitis (n = 63)	*p*-Value
Age (years)—median (*p* 25–75)	42 (35–49)	60 (52–68)	<0.001
Gender female—n (%)	37 (66.1)	39 (61.9)	0.70
Diabetes mellitus—n (%)	0	5 (7.9)	0.06
Arterial hypertension—n (%)	3 (5.4)	19 (30.2)	0.001
Cardiovascular disease—n (%)	3 (5.4)	4 (4.3)	0.12
Hypercholesterolemia—n (%)	2 (3.6)	2 (3.2)	0.99
Rheumatoid arthritis—n (%)	1 (1.8)	4 (6.3)	0.37
Systemic lupus erythematosus—n (%)	0	1 (1.6)	0.99
Body mass index (kg/m^2^)—median (*p* 25–75)	24.9 (22.9–26.9)	24.8 (22.7–28.4)	0.94
Obesity—n (%)	7 (12.7)	9 (14.3)	0.99
Coffee—n (%)	48 (85.7)	55 (87.3)	0.99
Never smoker—n (%)	43 (76.8)	27 (42.9)	<0.001
Tea—n (%)	5 (8.9)	3 (4.8)	0.47
Alcohol—n (%)	26 (46.4)	31 (49.2)	0.86
Immunosuppressive therapy—n (%)	1 (1.8)	4 (6.3)	0.37
Radiotherapy—n (%)	0	3 (4.8)	0.25
Methotrexate—n (%)	1 (1.8)	0	0.47
Malondialdehyde (nmol/mL)—median (*p* 25–75)	0.71 (0.60–0.83)	0.84 (0.69–0.99)	<0.001

The comparison between subject groups in categorical variables was carried out using the Chi-square test, while continuous variables were analyzed using the Mann–Whitney U-test.

**Table 3:** Multiple logistic regression analyses to determine factors associated with periodontitis.

Variables	Odds Ratio	95% Confidence Interval	*p*-Value
Age (years)	1.108	1.054–1.165	<0.001
Arterial hypertension (yes vs. no)	1.993	0.405–9.240	0.41
Never smoked (yes vs. no)	0.401	0.147–1.097	0.08
Salivary malondialdehyde levels > 0.77 nmol/mL (yes vs. no)	3.384	1.266–9.047	0.02

## Data Availability

The raw data supporting the conclusions of this article will be made available by the authors on request.
